# Supplementary figures and images for: An experimental-mathematical approach to predict tumor cell growth as a function of glucose availability in breast cancer cell lines
Source: PLoS One. 2021 Jul 13;16(7):e0240765. doi: 10.1371/journal.pone.0240765 (PMC8277046; doi:10.1371/journal.pone.0240765)

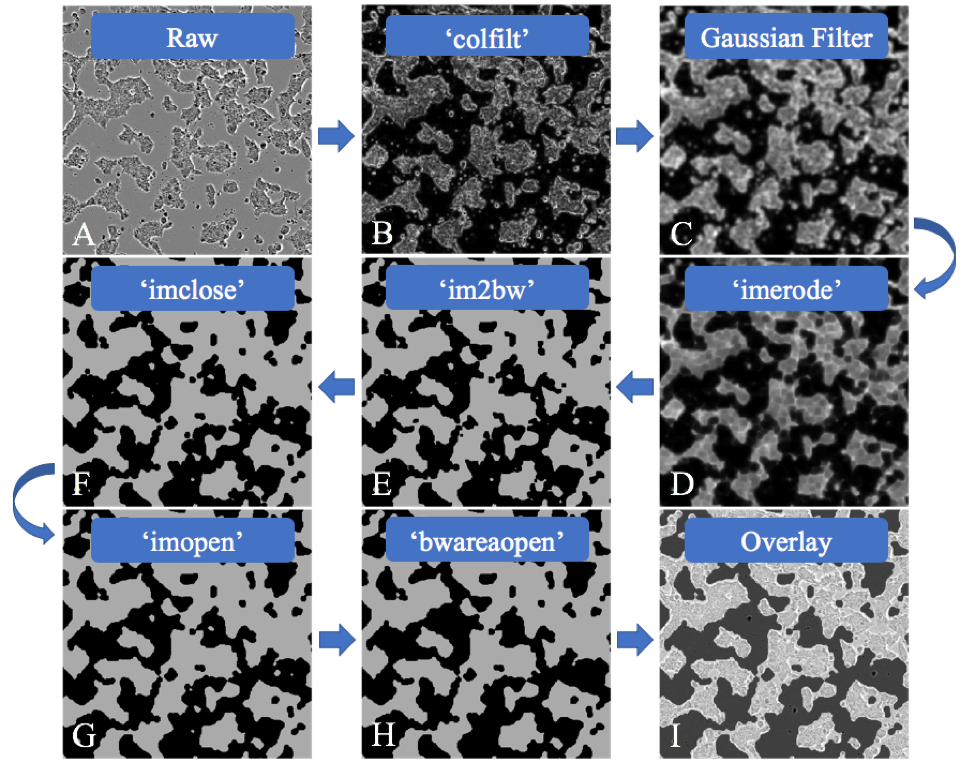

Supplement: S1 Fig — The size of the whole well image is 2400 x 2400 pixels. Here we present a window of 400 x 400 pixels from an example image. Panel A: raw image of BT-474 cells; Panel B: image post ‘colfilt’; Panel C: image post the Gaussian filter; Panel D: image post ‘im2bw’; Panel E: image post ‘imerode’; Panel F: image post ‘imclose’; Panel G: image post ‘imopen’; Panel H: image post ‘bwareaopen’, the final cell mask; Panel I: overlay of raw image and the cell mask for BT-474 cells. (TIF) [file pone.0240765.s001.tif]

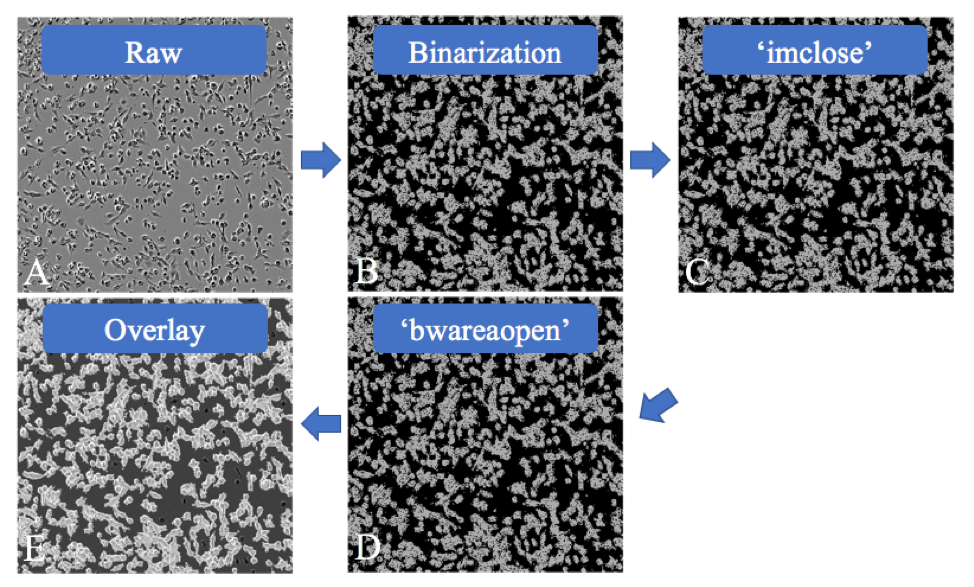

Supplement: S2 Fig — The size of the whole well image is 2400 x 2400 pixels. Here we present a window of 400 x 400 pixels from an example image. Panel A: raw image of MDA-MB-231 cells; Panel B: image post binarization; Panel C: image post ‘imclose’; Panel D: image post ‘bwareaopen’, the final cell mask; Panel E: overlay of raw images and the cell mask for MDA-MB-231 cells. (TIF) [file pone.0240765.s002.tif]

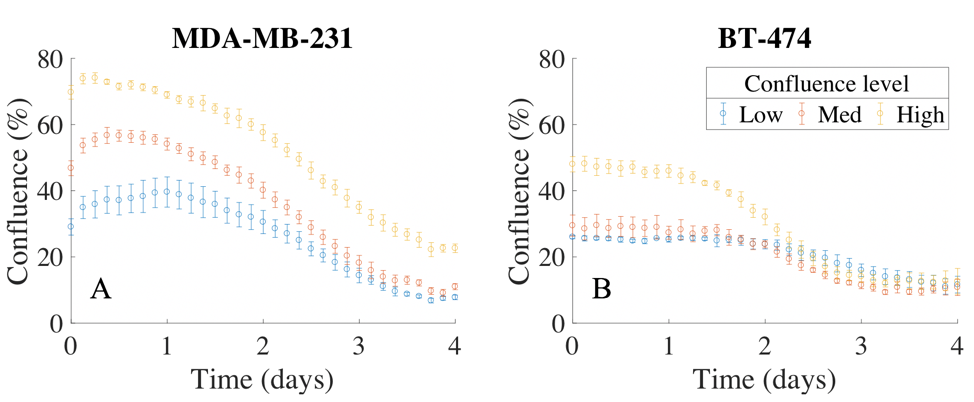

Supplement: S3 Fig — Tumor cells may keep proliferating for some time even in a glucose free medium, even up to 24 hours for MDA-MB-231 (Panel A). The proliferation in glucose free medium is not observed for BT-474 (Panel B). (TIF) [file pone.0240765.s003.tif]

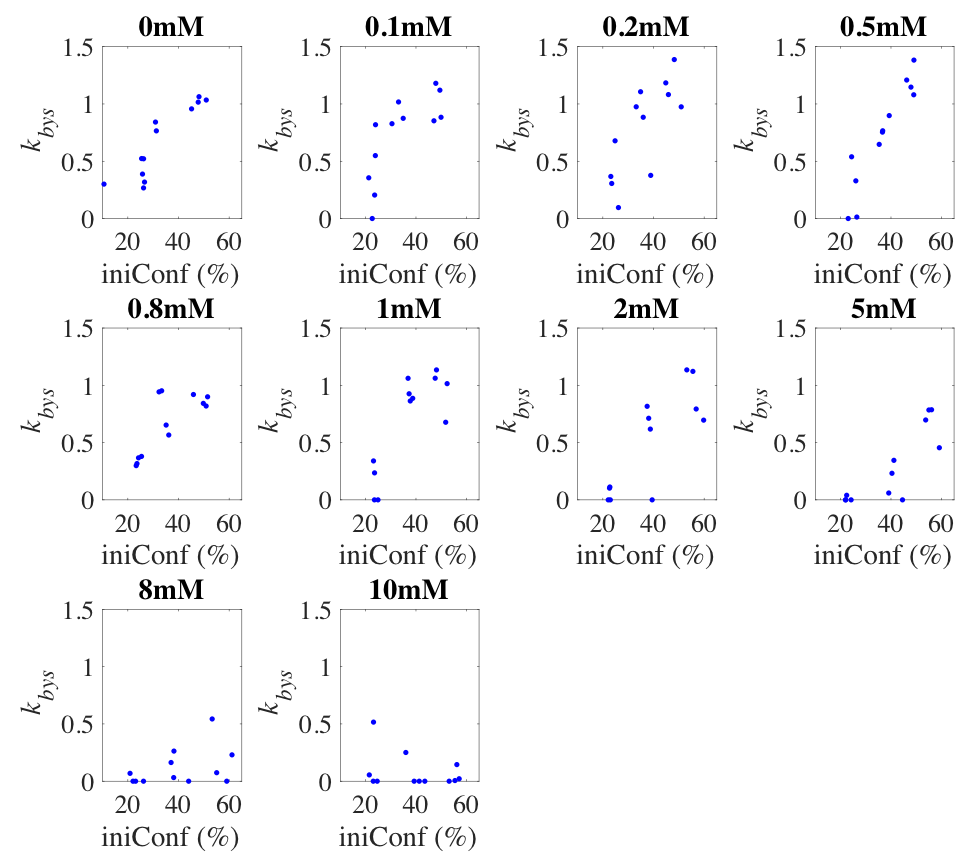

Supplement: S4 Fig — Each subtitle indicates the initial glucose concentration. For a given initial glucose level, kbys increases with initial confluence. For 8 of 10 initial glucose levels tested (0, 0.1, 0.2, 0.5, 0.8, 1, 2, and 5 mM, the bystander effect death rate is positively correlated with initial confluence, with correlation coefficients all > 0.74 (p < 0.01). For the highest two initial glucose levels (8 and 10 mM), there is no significant correlation between the bystander effect death rate and the initial confluence (p > 0.1). (TIF) [file pone.0240765.s004.tif]

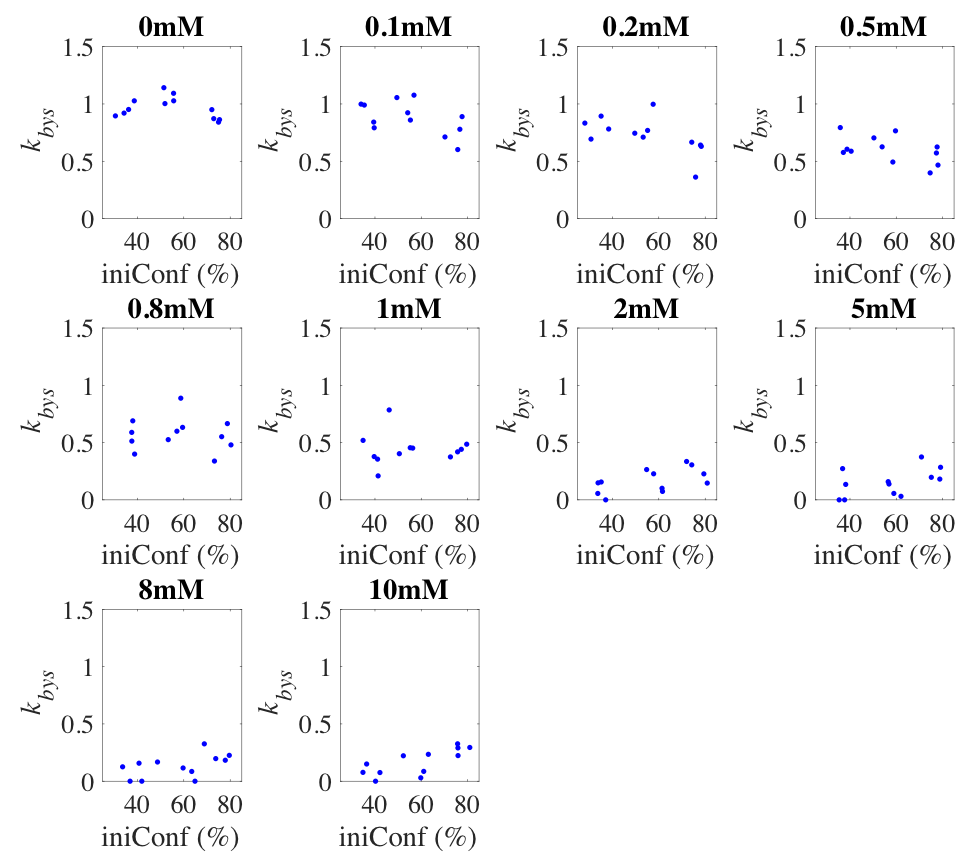

Supplement: S5 Fig — Each subtitle indicates the initial glucose concentration. There is no significant correlation between the bystander effect death rate and the initial confluence (p > 0.1). (TIF) [file pone.0240765.s005.tif]

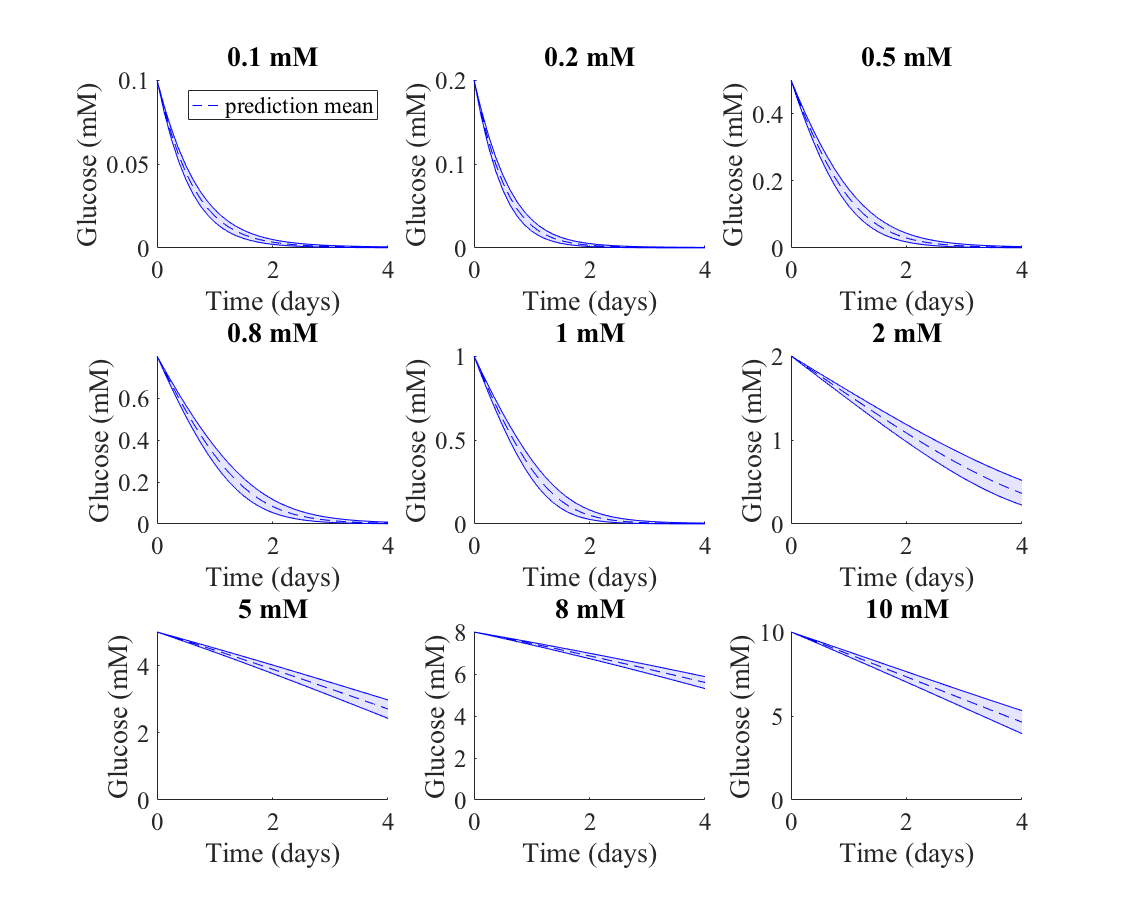

Supplement: S6 Fig — Example model predictions of glucose levels from one validation set of BT-474 cells. The average glucose levels from predictions are shown as dashed lines, with the 95% confidence intervals shown as shaded regions between the solid curves. The initial glucose level is shown above each plot. Please note the scales of vertical axis in each panel are different to better visualize the change of glucose levels. (TIF) [file pone.0240765.s006.tif]

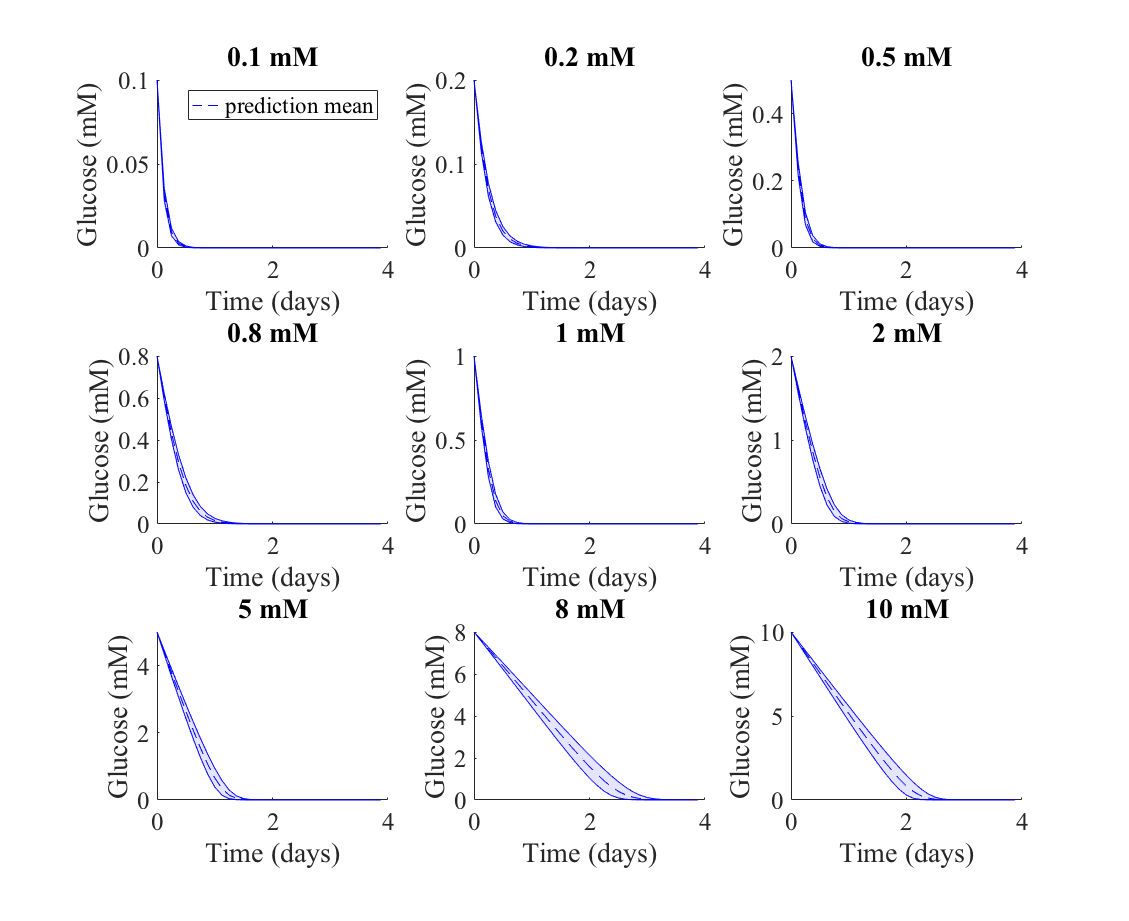

Supplement: S7 Fig — Example model predictions of glucose levels from one validation set of MDA-MB-231 cells. The average glucose levels from predictions are shown as dashed lines, with the 95% confidence intervals shown as shaded regions between the solid curves. The initial glucose level is shown above each plot. Please note the scales of vertical axis in each panel are different to better visualize the change of glucose levels. (TIF) [file pone.0240765.s007.tif]
