# Supplementary material for: An experimental-mathematical approach to predict tumor cell growth as a function of glucose availability in breast cancer cell lines
Source: PLoS One. 2021 Jul 13;16(7):e0240765. doi: 10.1371/journal.pone.0240765 (PMC8277046; doi:10.1371/journal.pone.0240765)
Supplement: S1 Table — Model 2 is the complete model described by Eq [1]–[5]. In Model 1, all the terms involving kbys is removed, while in Model 3, any term involving kd is removed. In the first calibration, the measured live and dead tumor cell time courses are independently fit to the model to produce separate estimates for each model parameter. In the second calibration, all the parameters are treated as global parameters. In the third calibration, kbys is considered as a local parameter while the other parameters (kp, kd, and v) are treated as global parameters. (DOCX) [file pone.0240765.s008.docx]

| Cell Line | Calibration | Model 01 | Model 02 | Model 03 |
| --- | --- | --- | --- | --- |
| BT-474 | First | 60.85 | 19.87 | 31.62 |
|  | Second | 129.25 | 84.27 | 136.08 |
|  | Third | - | -59.24 | - |
| MDA-MB-231 | First | 60.82 | 13.74 | 29.57 |
|  | Second | 136.30 | 99.15 | 137.39 |
|  | Third | - | -48.55 | - |
