# Supplementary material for: An experimental-mathematical approach to predict tumor cell growth as a function of glucose availability in breast cancer cell lines
Source: PLoS One. 2021 Jul 13;16(7):e0240765. doi: 10.1371/journal.pone.0240765 (PMC8277046; doi:10.1371/journal.pone.0240765)
Supplement: S2 Table — Xmodel,ij is the number of live or dead cells of well j at timepoint i calculated from the model, Xdata,ij is the number of live or dead cells of well j at timepoint i from the measured data, t is the total number of timepoints, w is the total number of wells, and tend is the last timepoint at the end of experiment (EoE). (DOCX) [file pone.0240765.s009.docx]

| Evaluation Variables | Matrices |
| --- | --- |
| Mean % Error |  |
| % Error EoE |  |
| Mean Error |  |
| Error EoE |  |
| Uncertainty |  |
