# Supplementary material for: An experimental-mathematical approach to predict tumor cell growth as a function of glucose availability in breast cancer cell lines
Source: PLoS One. 2021 Jul 13;16(7):e0240765. doi: 10.1371/journal.pone.0240765 (PMC8277046; doi:10.1371/journal.pone.0240765)
Supplement: S3 Table — The table shows the relative contributions of each term of Eq [1] on days 0, 2, and 4 for different initial confluences and three initial glucose concentrations (0, 1, and 10 mM). (DOCX) [file pone.0240765.s010.docx]

| Initial confluence (%) | Initial Glucose (mM) | Time (day) | Relative contributions (%) | | |
| --- | --- | --- | --- | --- | --- |
|  |  |  | Logistic growth | Death due to glucose depletion | Death due to bystander effect |
| 26.6 | 0 | 0 | 88.4 | 0.0 | 11.6 |
|  |  | 2 | 1.3 | 61.7 | 37.0 |
|  |  | 4 | 0.0 | 42.8 | 57.2 |
| 51.0 | 0 | 0 | 68.4 | 0.0 | 31.6 |
|  |  | 2 | 0.4 | 22.8 | 76.8 |
|  |  | 4 | 0.0 | 12.1 | 97.9 |
| 23.6 | 1 | 0 | 95.0 | 0.0 | 5.0 |
|  |  | 2 | 86.6 | 5.4 | 7.9 |
|  |  | 4 | 47.2 | 36.9 | 15.9 |
| 47.6 | 1 | 0 | 66.2 | 0.0 | 33.8 |
|  |  | 2 | 17.3 | 9.3 | 73.3 |
|  |  | 4 | 1.2 | 12.7 | 86.1 |
| 21.5 | 10 | 0 | 96.2 | 0.0 | 3.8 |
|  |  | 2 | 96.0 | 0.2 | 3.8 |
|  |  | 4 | 95.8 | 0.2 | 3.9 |
| 55.5 | 10 | 0 | 99.8 | 0.0 | 0.2 |
|  |  | 2 | 99.3 | 0.5 | 0.2 |
|  |  | 4 | 98.8 | 0.9 | 0.2 |
