# Supplementary material for: An experimental-mathematical approach to predict tumor cell growth as a function of glucose availability in breast cancer cell lines
Source: PLoS One. 2021 Jul 13;16(7):e0240765. doi: 10.1371/journal.pone.0240765 (PMC8277046; doi:10.1371/journal.pone.0240765)
Supplement: S4 Table — The table shows the relative contributions of each term of Eq [1] on days 0, 2, and 4 for different initial confluences and three initial glucose concentrations (0, 1, and 10 mM). (DOCX) [file pone.0240765.s011.docx]

| Initial confluence (%) | Initial Glucose (mM) | Time (day) | Relative contributions (%) | | |
| --- | --- | --- | --- | --- | --- |
|  |  |  | Logistic growth | Death due to glucose depletion | Death due to bystander effect |
| 30.6 | 0 | 0 | 58.8 | 0.0 | 41.2 |
|  |  | 2 | 1.1 | 9.9 | 89.1 |
|  |  | 4 | 0.0 | 5.4 | 94.6 |
| 75.0 | 0 | 0 | 55.3 | 0 | 44.7 |
|  |  | 2 | 0.9 | 13.2 | 85.9 |
|  |  | 4 | 0.0 | 6.4 | 93.6 |
| 40.0 | 1 | 0 | 81.8 | 0.0 | 18.2 |
|  |  | 2 | 50.2 | 14.2 | 35.6 |
|  |  | 4 | 0.5 | 30.0 | 69.9 |
| 77.3 | 1 | 0 | 69.7 | 0.0 | 30.3 |
|  |  | 2 | 2.6 | 36.9 | 60.5 |
|  |  | 4 | 0.0 | 20.9 | 79.1 |
| 42.2 | 10 | 0 | 95.0 | 0.0 | 5.0 |
|  |  | 2 | 94.5 | 0.1 | 5.4 |
|  |  | 4 | 93.9 | 0.1 | 5.9 |
| 75.9 | 10 | 0 | 76.2 | 0.0 | 23.8 |
|  |  | 2 | 65.7 | 0.1 | 34.2 |
|  |  | 4 | 55.0 | 0.7 | 44.3 |
