# Supplementary material for: An experimental-mathematical approach to predict tumor cell growth as a function of glucose availability in breast cancer cell lines
Source: PLoS One. 2021 Jul 13;16(7):e0240765. doi: 10.1371/journal.pone.0240765 (PMC8277046; doi:10.1371/journal.pone.0240765)
Supplement: Attachment — Submitted filename: Yankeelov_PONE_Nov2020.pdf [file pone.0240765.s012.pdf]

Title: An experimental-mathematical approach to predict tumor cell growth as a function of glucose availability in breast cancer cell lines
Authors: Yankeelov et al
Your ref: PONE-D-20-30580

Summary: The authors use 3 related mathematical models to analyse time course data from experiments involving 2 breast cancer cell lines, in which the cells are cultured at different glucose levels and plated at different levels of confluence. The study is a great example of how experimental and mathematical approaches can be combined to increase understanding of biological systems. I am generally supportive of the work but, as outlined below, I have several issues with the modelling assumptions. Additionally, in several places, more details are needed so the reader is clear on the methodology. Finally, the main results and/or findings of the paper could be more clearly stated (and related back to the original goals of the study). For these reasons, I recommend a major revision of the article.

Detailed comments:

- Figure 1 (legend) and section 2.8: please clarify how the data were divided into training and validation subsets: different glucose levels and/or confluence levels or experimental replicates?
- Section 2.1: The experiments were performed in 2D. Could the authors comment on how relevant these results will be for 3D growth, in vitro and/or in vivo?
- Section 2.4 (equations (1)-(3)): The model should be better justified. For example, why don't the dead cells compete for space with the live cells? Given that they are adherent to the substrate, isn't competition with dead cells important? Also, why is there no loss term for dead cells? Given that the experiments run over a 4-day period, is it justifiable to neglect degradation of dead cells? For example, is it possible that dead cells become detached from the substrate and are then removed from the system? Better justification for the bystander effect is needed: what might this represent? The accumulation of lactate from cell metabolism?
- Section 2.4 (equations (4) and (5), and lines 217-224): please can you explain more clearly what the tanh(t) term represents? A time delay in the duration of mitosis would be modelled differently (eg $S_d(G(t\text{-}tau))$ where tau is the time for mitosis). Does the tanh(t) term represent the time it takes for the cells to adjust to their environment after plating? How well does your model perform if you neglect this term?
- Page 11 (lines 223-224): what is the relevance of the identity '$S_d + S_p = 1$?'
- Page 11 (lines 227-232): please clarify what processes are included in the 2 reduced models – Model 2 neglects cell death due to bystander effect, and model 3 neglects cell death due to glucose deprivation but retains cell death due to the bystander effect?
- Page 12 (lines 245-246): please clarify the meaning of this sentence – did you fit your model to the live cell data only and then fit it separately to the dead cell data?
- Page 12 (lines 247-258): what does this mean? What was the logic for doing this? Why should model parameters depend on initial conditions? Does this mean that your original model is overly simplistic and should be revised? This approach needs to be carefully justified, and arguments against more alternative options included.

- Page 13 (lines 269-271): please can you clarify what you do here? You average the data from the first 3 time points? What is the justification for doing this? How sensitive are your results (esp parameter estimates) to the choice of the initial conditions?
- Section 2.7: I understand the results but, as mentioned above, they suggest that the model needs revision. We are told you 'found' a relationship between k_{bys}, the initial number of tumor cells and the initial glucose level – what other relationships did you try? Did you consider alternative, more physically-based models? You introduce 2 extra parameters (see equation (8)). How many extra parameters would be needed for a more physically-based model extension, where parameters do not depend on initial conditions? (eg introduce an additional variable for lactate levels, assume lactate production occurs at a rate proportional to glucose consumption, and lactate degradation at rate proportional to lactate levels, then cell death (your bystander term) is an increasing function of lactate levels – this extension would involve 2 additional parameters – did you try this?)
- Section 3.1: please consider presenting the results for the 2 cell types in a table – this would be easier to digest than reading from the text.
- Section 3.2: Please can you clarify that no time course data on glucose levels was collected? It would be helpful to see what the model simulations predict for this hidden variable. It would be great, for example, to see how glucose levels are predicted to fall (and possibly how levels of waste products, eg lactate, increase) during the time course of the experiments.
- Section 3.2: are theta and G_{min} global variables? How were they determined?
- Section 3.2 (line 390): do you have any idea why the error for dead cells is so large? (see earlier comments re model equations for possible explanations for this discrepancy)
- Section 3.3: could you comment on the relative contributions that the different physical terms in the governing equations have on the growth dynamics (this is difficult to infer from the way that the parameter estimates are presented). For example, does cell proliferation dominate? How significant is the bystander effect? I appreciate that the importance of the different processes will change over time, but it would be great to have some sense of their relative contributions (perhaps at different time-periods – beginning, middle and end of experiments?).
- Section 4 (lines 593-609): these are good points, well made.
- Section 5: what is the main conclusion/finding of the work? What new insight has been gained? Which model is most suitable? Why?
- General comment: the authors might consider using their model(s) to perform synthetic studies which could aid experimental design (see for example C Kreutz and J Timmer, FEBS Journal, 2009). Such studies might reveal the value of collecting glucose data at specific time points during the experiment, not necessarily at every time point.
