# Supplementary material for: An experimental-mathematical approach to predict tumor cell growth as a function of glucose availability in breast cancer cell lines
Source: PLoS One. 2021 Jul 13;16(7):e0240765. doi: 10.1371/journal.pone.0240765 (PMC8277046; doi:10.1371/journal.pone.0240765)
Supplement: Attachment — Submitted filename: JY_Response to Reviewers.docx [file pone.0240765.s013.docx]

**Manuscript #PONE-D-20-30580**

We thank the reviewers for their careful review of our manuscript and for providing such constructive criticism. We have taken their suggestions and criticisms seriously, and have revised the manuscript to address their concerns. As a result of their feedback, we believe the quality and clarity of our manuscript has substantially improved. Detailed responses to each item are provided below. In our responses below, for clarity, we have referred to Review x, Comment y as ‘*Rx.y*’.

**Editor’s Comments:**

*Thank you for submitting your manuscript to PLOS ONE. After careful consideration, we feel that it has merit but does not fully meet PLOS ONE’s publication criteria as it currently stands. Therefore, we invite you to submit a revised version of the manuscript that addresses the points raised during the review process. In particular, the proposed experimental-computational approach is very interesting but the modelling assumptions need to be justified and detailed more. We would also like to encourage you to include more details on data availability.*

Response:

We sincerely thank the Editor for their comments on the initial submission. As indicated below (and in the revised manuscript), we have worked hard to address each point raised by the reviewers.

We have made both our data and code fully available for download on figshare.com. We have updated the data availability to include a link to the code.

**Specific review questions:**

1. Is the manuscript technically sound, and do the data support the conclusions?

**Reviewer #1 and #2:** Partly

Response:

We believe the reviewers’ major technical concerns were about model assumptions and we have worked our best to address these issues. In particular, we have justified the proliferation term, the death term due to glucose depletion and bystander effect, the use of the hyperbolic tangent function of time, and the approach of having both global (cell line specific) and local (initial condition dependent) parameters in our model. We also added more explanation and justification of the methodology to make it clearer (e.g., the sampling approach for training sets is discussed in more detail). All of these details have been included in the revised Materials and Methods section.

Regarding the question of the data supporting our conclusions, our primary conclusions are that we have developed and validated an experimental-mathematical approach that is capable of accurately predicting how glucose availability influences tumor cell proliferation. With the accuracy and error reported, we believe our model performed well at predicting tumor cell growth.

We hope that the further details (mentioned in the previous paragraph) help to support our methodology to reach the conclusions we highlight in the manuscript.

Additionally, Reviewer 1 stated that the equations were unreadable. We have attempted to address this issue in the revised manuscript by confirming the readability of the equations with the manuscript in .docx format and in .pdf format. We also confirmed the readability in both the Mac and Windows operating systems. Please know that this was only a display issue in the pdf and did not affect the application of our approach or our conclusions.

2. Has the statistical analysis been performed appropriately and rigorously?

**Reviewer #1:** Yes

Response:

We thank the reviewer for their support.

**Reviewer #2:** N/A

Response:

N/A

3. Have the authors made all data underlying the findings in their manuscript fully available?

**Reviewer #1:** Yes

Response:

Please see our response to Reviewer 2 on this point.

**Reviewer #2:** No

Response:

We thank the reviewer for this comment and have made both our data and code fully available for download on figshare.com. We have updated the section on data availability to include a link to the code.

4. Is the manuscript presented in an intelligible fashion and written in standard English?

**Reviewer #1 and #2:** Yes

Response:

We thank the reviewers for their support.

Reviewer 1 individual comments

*R1.0) This is an interesting paper focusing on fitting different models to the experimental data on the temporal change of the number of live and dead tumor cells under different initial glucose concentrations and seeding densities. However, I have number of concerns regarding the modelling, which I believe have to be addressed.*

Response:

We sincerely thank the reviewer for their support and have worked to address all of their concerns.

*R1.1) Unreadable mathematical expressions: Practically all equations ([1]-[6]) in the manuscript are unreadable , as many squares and rectangles appear instead of signs +,-, and =, and possibly also brackets. As a consequence, the rationale behind those equations cannot be checked, which makes it impossible for me to give my full judgement of the models used. This needs to be corrected.*

Response:

We apologize for this unfortunate situation and are not sure how or why it happened. In the pdf we uploaded for the first submission, all the equations display as they should. Since Reviewer 2 did not mention this issue, we are wondering if there is a display issue or a Mac to PC issue? Regardless, before uploading we checked the readability of the equations in the manuscript in both .docx and .pdf formats. We also confirmed the readability on both the Mac and Windows operating systems. Hopefully, the equations are readable now.

*R1.2) Unmotivated manipulation of death rates as opposed to growth rates: I do not understand why when you experimentally manipulate the glucose concentration which should contribute to faster proliferation, your models do not differ in their growth rate, but instead in their death rate. Please explain or switch to more standard interpretation where glucose contributes to faster proliferation of cells.*

Response:

We apologize for the lack of clarity on this point. In our approach, the proliferation (growth) rates and death rates should be considered as the maximum rates possible, while the real-time proliferation (growth) rates or death rates due to glucose depletion are modified by the state functions (Eqs. [4] and [5]). According to Eq. [5], as *G*(*t*) ➝ ∞, *S_p_*(*G*(*t*)) ➝ 1, the growth rate is maximized. In contrast, as *G*(*t*) ➝ 0, *S_p_*(*G*(*t*)) ➝ 0, the growth rate is minimized. In another words, more glucose contributes to faster proliferation of cells. Similarly, according to Eq. [4], the death rate due to glucose depletion is maximized as *G*(*t*) ➝ 0 and minimized as *G*(*t*) ➝ ∞. In summary, our models accounts for changes in both the growth and death rates due to glucose depletion depending on real-time glucose concentrations. We have included these comments after Eqs. [4] and [5] are first introduced.

*R1.3) Unmotivated simplification of the model by Mendoza-Juez et al: It is unclear why the authors do not use 2 phenotypes as Mendoza-Juez et al. but instead assume that all cells use anaerobic glycolysis. Is that inherent to the cell lines used? Is it 100% sure that no cells use oxidative phosphorylation? If not, why isn't it better to estimate the subpopulations pf Mendoze-Juez as opposed to removing one type? (Here I note that my problem with this may be a consequence of inability to read the equations, as mentioned in 1., thus correction of that problem as asked in 1. may help me to understand the simplification better)*

Response:

We thank the reviewer for their insight into the simplification of the model by Mendoza-Juez *et al*. As was noted in the Mendoza-Juez *et al*. study, it was assumed that the two phenotypes (i.e., oxidative cells and glycolytic cells) shared the same genotype, and thus each can be understood as a fraction of the total cell number—or as a fraction of each cell’s metabolism (a single cell may use a certain fraction of glucose for anaerobic glycolysis, while the remaining cells may use oxidative phosphorylation). Thus, the total cell growth is the sum of the growth of both populations, and the total proliferative rate can be seen as a weighted average of oxidative cells proliferation rate and glycolytic cells proliferation rate, weighted by the fraction that each metabolic phenotype contributes to the overall cell number. Specifically, when we consider the fraction as a fraction of each cell’s metabolism, there can be more than two states. Some cells may use only oxidative phosphorylation while some cells may use only anaerobic glycolysis, while other cells may have states between complete oxidative phosphorylation and complete anaerobic glycolysis. Therefore, assuming only two populations is also a simplification of all the possible states. In our approach we did not assume that all cells use anaerobic glycolysis, but rather include all possible states between complete oxidative phosphorylation and complete anaerobic glycolysis. This is most easily seen in Eq. [1], where we treat the proliferation (growth) rate as the maximum rates possible under any metabolism. When the fraction of oxidative phosphorylation and anerobic glycolysis varies in response to different glucose levels, the modified real-time proliferation (growth) rate represents a weighted-average of proliferation rate under complete oxidative phosphorylation and proliferation rate under complete anerobic glycolysis. Therefore, we decided to focus on the overall cell population, rather than each cell’s individual metabolic phenotype. In our model the proliferation rate can be considered as an average of all cells possible proliferative activity (similarly for the death rate). Our simplification avoided the need to monitor the conversion between the two phenotypes as well as the fractional change over time. This approach has both experimental and computational advantages. Experimentally, we are not forced to develop a method that is capable of making serial, non-destructive measurements of metabolic activity of the same cells over time. Computationally, this approach reduces the number of parameters that we would need to calibrate from experimental data. We have summarized these comments in the revised Discussion section.

*R1.4) Strong dependence on the results on the percentage of training and validation data: The authors report that with the selected model, the achieved accuracy (defined as the fraction of
measured data that fell within the 95% confidence intervals of the predicted growth curves) is 77.2 ± 6.3% and 87.2 ± 5.1% for live BT-474 and MDA-MB-231 cells, respectively. This accuracy will surely depend on the percentage of data used for training/validation. Assuming that there are reasons for having the model form the authors use (see concerns I express in my previous points), could the authors explain why exactly 75% of data are used for training (and not more or less)? And if not, could they perform sensitivity analysis with respect to this percentage? Also, I do not perceive 77.2% as particularly high. Could the authors comment why they think their model outperforms other, perhaps more standard models when fitting the data?*

Response:

We appreciate the comment on the approach and results of training and validation. A 75/25 split of the data into a training set and validation set is one of the common practices for Monte Carlo cross validation. (We have added some influential references where the same ratio was employed [1,2].) We agree that there could be other options for data splitting, but there is no “gold-standard” because of the trade-off in bias and variance for the choice of the splitting ratio, specifically, the larger the size of the training set, the lower the bias and the higher the variance. When we increase the size of the training set, we will be able to calibrate the model better, but it also comes with a higher chance of overfitting, reducing the generalization ability of the model. The goal of the training and validation is not to find the percentage (or the size of the training set) that gives the best prediction, but test the performance of the model given different data samples. As shown in Table 5, the model predicted the cell confluence time courses with mean error and error at the end of the experiment all below 3%, for both live and dead cells in both cell lines, with model calibration over 50 different training sets. Thus, we feel the model performs well at predicting tumor cell growth.

We agree that 77.2% is not especially high. But please note that this accuracy was *over the entire-time course* of the growth curve. Perhaps more importantly, the accuracy we defined was not the only criteria we used to evaluate the prediction. In addition to the accuracy, we also computed the averages of the RSS, mean percent error over the time course, percent error at the end of experiment, mean error over the time course, and error at the end of experiment; see S1 Table of the Supporting Information. In particular, the mean error between the predicted and measured confluence over the whole time course of the live and dead cells was below 2% for both cell lines, while the error at the end of the experiment was below 3% for both cell lines. Considering both the error and the accuracy, we believe our model is performing quite well at predicting the tumor cell growth.

*R1.5) I would be more modest in statements like this: "To the best of our knowledge, this represents the first time these important cellular parameters have been quantified within a rigorous modeling framework." I know of many studies where cellular parameters are quantified with a modeling framework (eg Kaznatcheev et al, Nature Eco Evo 3(3), 2019; or works by Brady and Enderling....), and I am sure the authors know many, too.*

Response:

We appreciate and agree with the review’s comment. We have removed the statements.

*R1.6) It is interesting to see a paper on Warburg effect which does not mention a large body of literature of game theoretical models that attempt to explain/quantify it, for example Archetti at al, Epstein et al. I would recommend adding these references.*

Response:

We appreciate the recommendation and have revised the manuscript with references on game theoretical models added.

*R1.7) "The major source of energy for most cancer cells comes from a high rate of glycolysis
followed by lactate fermentation in the cytosol, even in the presence of sufficient oxygen—a
phenomenon known as the Warburg effect [1,2]." - Are you sure of the word "most" here?*

Response:

We appreciate the comment and apologize for the lack of clarity on this point. Here the word “most” refers to the ascites cancer cells in Warburg’s work[3] when the Warburg effect was first discovered and defined, with 85% of the pyruvate converted to lactate[3,4]. We are not talking about most of the cancer types as the Warburg effect is not consistent across all cancer types. We have changed the word “most” to “many” in the revised manuscript.

Reviewer 2 individual comments

*R2.0) The authors use 3 related mathematical models to analyse time course data from experiments involving 2 breast cancer cell lines, in which the cells are cultured at different glucose levels and plated at different levels of confluence. The study is a great example of how experimental and mathematical approaches can be combined to increase understanding of biological systems. I am generally supportive of the work but, as outlined below, I have several issues with the modelling assumptions. Additionally, in several places, more details are needed so the reader is clear on the methodology. Finally, the main results and/or findings of the paper could be more clearly stated (and related back to the original goals of the study). For these reasons, I recommend a major revision of the article.*

*It is not clear to me that the authors have made their data available. I would encourage them to include details explaining how people can access the data and use it to perform their own analysis of the data.*

Response:

We sincerely thank the reviewer for their support and have worked to address all of their criticisms. We have made both our data and code fully available for download on figshare.com. We have included links to both the data and code in the revised manuscript.

*R2.1) Figure 1 (legend) and section 2.8: please clarify how the data were divided into training and validation subsets: different glucose levels and/or confluence levels or experimental replicates?*

Response:

We sincerely apologize for the lack of clarity on this point. The 120 pairs (each “pair” in our experiments consisted of a confluence time course for live cells and a confluence time course for dead cells from the same well) of confluence time courses were numbered from 1 to 120. In each round of training, a random number generator (Matlab function ‘randperm’) was used to randomly select 90 integers from the integers 1 to 120 without repeats. The confluence time courses with the corresponding number were used as the training set (75% of the data), while the remaining 30 sets of confluence time courses were used as the validation set (25% of the data). We have revised the Section 2.8 of the manuscript with these details on how the data were divided into training and validation subsets.

*R2.2) Section 2.1: The experiments were performed in 2D. Could the authors comment on*

*how relevant these results will be for 3D growth, in vitro and/or in vivo?*

Response:

We thank the reviewer for raising this important point. In 2D cell culture, BT-474 cells form clusters, while elongated MDA-MB-231 cells do not form clusters. (The different morphologies of these two cell lines could be one of the reasons for their different growth profiles.) However, in 3D cell culture, BT-474 cells form colonies with round borders, while MDA-MB-231 cells present an invasive phenotype with stellate projections that often bridge multiple cell colonies [5]. BT-474 cells develop dense multicellular spheroids (MCSs) in 3D cell culture, while MDA-MB-231 cells develop only loosely aggregated MCSs [6]. These different architectures can lead to different microenvironments for the tumor cells, resulting in a non-uniform distribution of nutrients like glucose and oxygen. For example, hypoxic areas can be observed inside the dense 3D-MCSs from the BT-474 cell lines, but not in the loosely aggregated MCSs from the MDA-MB-231 cells or cells in 2D-culture [6]. This suggests a higher fraction of glycolysis for BT-474 cells compared to MDA-MB-231 cells, which indicates that we need to include cell line dependent parameters for nutrient diffusion, as the diffusion rate can depend on cellular density and tumor architecture. In addition, as hypoxia has been reported to cause cancer cell dormancy in the G0 phase [7], a reduced proliferation rate can be expected for BT-474 cells. Thus, when applying our modeling approach to 3D data, with more complicated microenvironments and tumor architectures, we would likely need to design new experiments to characterize the spatial distribution of the cells and nutrients. We have summarized the above points in the revised Discussion section.

*R2.3) Section 2.4 (equations (1)-(3)): The model should be better justified. For example, why don’t the dead cells compete for space with the live cells? Given that they are adherent to the substrate, isn’t competition with dead cells important? Also, why is there no loss term for dead cells? Given that the experiments run over a 4-day period, is it justifiable to neglect degradation of dead cells? For example, is it possible that dead cells become detached from the substrate and are then removed from the system? Better justification for the bystander effect is needed: what might this represent? The accumulation of lactate from cell metabolism?*

Response:

We appreciate the comment and have revised the manuscript with an updated justification of the model. We agree there is some competition for space between living and dead cells, and respectfully note that this effect is coupled into the last term on the right-hand side of Eqs. [1] and [2]. The term in the parenthesis describes the fraction of all cells comprised of dead cells, and accounts for the competition between live and dead cells. In particular, the higher the fraction of dead cells, the greater the competition is and the more this term contributes to the total cell death.

We agree it is possible that the dead cell become detached from the bottom of the plate and are removed from the system. However, we found through our experiments that it typically takes longer than 4 days for these cells to detach from the bottom of the plate. Therefore, we decided to use data only from the first 4 days and neglect the loss of dead cells from the system.

The motivation for incorporating the bystander affect is as follows. We noticed that the term describing the death due to glucose depletion was not sufficient to capture all the death observed in the experimental data. Considering the competition for space between the dead (prior to detaching) and live cells and, therefore, the potential cytotoxicity from the dead to the live cells, we introduced an extra term to account for this bystander effect. It represented the bulk phenomenon that dead cells can release factors, which may be sensed by the remaining live cells, and potentially induce cell death [8,9]. We note that the bystander effect is multi-factorial as it involves multiple mechanisms, either mediated by GJIC (gap junction intercellular communication) capacity [10–12] or soluble factors including apoptosis inducing signals [13], extracellular vesicles [14], or oxidizing diffusive factors [15]. We have included these comments in the revised Discussion section to provide further justification for employing the bystander effect (which is first introduced in Section 2.4).

*R2.4) Section 2.4 (equations (4) and (5), and lines 217-224): please can you explain more clearly what the tanh(t) term represents? A time delay in the duration of mitosis would be modelled differently (eg S_d_(G(t-tau)) where tau is the time for mitosis). Does the tanh(t) term represent the time it takes for the cells to adjust to their environment after plating? How well does your model perform if you neglect this* term?

Response:

We apologize for the lack of clarity on this point and have worked to address this in the revised manuscript. The hyperbolic tangent function is frequently used in modeling of biological systems to represent a switch function that enables the smooth transition (as opposed to an abrupt change described by, for example, the Heaviside function) from one “state” to another. The tanh(*t*) term represents the time it takes for the cells to adjust to their environment after the glucose concentration is changed. All the cells were cultured in medium (see Section 2.1 for details) with the same glucose concentration (we will refer to this medium as the “native” cell culture medium for the remainder of this response) after seeding (plating). Thus, all cell metabolism was initially driven by the same (native) glucose condition. After changing the media at the beginning of the experiment, the cells gradually switched to the second “state” where their metabolism were driven by the glucose concentrations in the media which were different than their native state. In our model, the tanh(*t*) term represents the time it takes for the cells to “switch” from the native state to the second “state”. Thus, the tanh(*t*) term represents the time for the cells to adjust to their environment after medium change, instead of after plating. The effect of the native cell culture media on the growth of the tumor cells would only exist at the earliest stages of the experiment, thereby making the tanh(*t*) function appropriate, while the term *S_d_*(*G*(*t-τ*)) has an effect throughout the entire experiment.

Regarding the effect of the tanh(*t*) term on the output of the model, when the term is removed from the model system for the BT-474 cells, the resulting model estimates *k_p_* 2.7% higher, *k_d_* 90.1% lower, and *v* 12.2% higher than the model that includes the tanh(*t*) term (i.e., the model presented in the manuscript). Similarly, for MDA-MB-231 cells, the model without the *tanh*(*t*) term estimates *k_p_* 6.9% higher, *k_d_* 100.0% lower, and *v* 13.1% lower than the model that includes the tanh(*t*) term (i.e., the model presented in the manuscript). For MDA-MB-231 cells, there was no contribution from death due to glucose depletion, with an estimated *k_d_* of 0 when the tanh(*t*) is removed.

*R2.5) Page 11 (lines 223-224): what is the relevance of the identity ‘S_d_ + S_p_ = 1?’*

Response:

We appreciate the comment on this point. Here we assumed that the tumor cells were either proliferating or dying; we have added this explanatory phrase in the revised manuscript.

*R2.6) Page 11 (lines 227-232): please clarify what processes are included in the 2 reduced models – Model 2 neglects cell death due to bystander effect, and model 3 neglects cell death due to glucose deprivation but retains cell death due to the bystander effect?*

Response:

We appreciate the comment and apologize for the lack of clarity on this point. The reviewer is exactly correct about this; i.e., model 2 neglects cell death due to the bystander effect, and model 3 neglects cell death due to glucose deprivation but retains cell death due to the bystander effect. We have added text to explicitly state these points after the models are first presented in the revised Materials and methods section.

*R2.7) Page 12 (lines 245-246): please clarify the meaning of this sentence – did you fit your model to the live cell data only and then fit it separately to the dead cell data?*

Response:

We apologize for the lack of clarity on this point. We fit the model to one confluence time course of live cells and the corresponding confluence time course of dead cells simultaneously. We conducted model fitting like this for all 120 pairs of living and dead confluence time courses to generate 120 sets of parameter estimates. We have attempted to clarify the description of the fitting approach in the revised Section 2.5.

*R2.8) Page 12 (lines 247-258): what does this mean? What was the logic for doing this? Why should model parameters depend on initial conditions? Does this mean that your original model is overly simplistic and should be revised? This approach needs to be carefully justified, and arguments against more alternative options included.*

Response:

We appreciate these comments and the opportunity to more fully explain our thought process. At the outset, we had no knowledge about the relationship (or, if there even was one) between model parameters and initial conditions. Thus, we began by fitting every parameter as a local parameter across all confluence time courses which provided the lowest error (the difference between model fitting and measured data), but came with a high possibility of overfitting. We then fit every parameter as a global parameter which provided the highest error, but least likely to overfit. We then systematically investigated a range of combinations of local and global parameters to achieve a balance between the fitting error and the risk of overfitting. We investigated the distributions of estimates of the local parameters in different combinations. We noticed that the estimates of *k_p_*, *k_d_*, and *v* tended to converge to similar values across replicates, suggesting they could be global parameters, while the estimates of *k_bys_* presented a wide distribution. It was only after we calibrated the model with *k_p_*, *k_d_*, and *v* as global parameters, and *k_bys_* as a local parameter, we discovered the dependence of *k_bys_* on the initial conditions. Since the initial conditions can drive the tumor cells into different metabolic states varying between complete oxidative phosphorylation and compete anerobic glycolysis, the parameters, as a representative of the metabolic states, can be dependent on the initial conditions. The higher *k_bys_* observed at the lower initial glucose levels (and higher initial confluences for BT-474 cells) for both cell lines, can be interpreted as an increase in competition between cells when fewer nutrients are available.

On the point about the model being overly simplistic, this is (of course) always a concern. However, we always attempt to select the most parsimonious model that is capable of describing the data with the least free parameters. If we were to extend the model to account for more phenomena, we would almost certainly require more measurements of different processes to be able to calibrate the model properly. In fact, we respectfully propose that our model may be quite general as it does not require information that might be cell line specific; therefore, it can potentially be calibrated to any other cell line for which the time course data is available. For example, the distribution of *k_bys_* does not depend on how we write or fit Eqs. [7] and [8], and therefore the approach can be applied to any cell line. As long as we have data that captures a range of initial conditions, we can calibrate the model and obtained estimates for both global and local parameters for new cell lines. From there we have the flexibility of exploring the relationship between the local parameters and the initial conditions (or even building a look-up table when it is hard to find the relationship), to determine the local parameters used for prediction given new initial conditions. It is certainly true we could revise the model by including Eq. [7] or Eq. [8] into Eqs. [1] and [2], but chose not to as we sought to achieve a more generalizable model. As it was shown in the results, this relationship may vary between different cell lines. Therefore, a model including this specific relationship may have difficulty being applied to all cell lines. Rather, multiple models would be needed and they would have to be tested against each cell line considered.

(Please also see our response below to R2.10 on this important point.)

*R2.9) Page 13 (lines 269-271): please can you clarify what you do here? You average the data from the first 3 time points? What is the justification for doing this? How sensitive are your results (esp. parameter estimates) to the choice of the initial conditions?*

Response:

We average the data from the first three time points and use the resulting value as the initial number of tumor cells present in the well. We assumed that there was very little change in cell number during the first three time points which corresponds to 6 hours. Therefore, we could use the average of the first three time points to improve our estimate of the initial cell number.

In regards to how this affected parameter values, for the BT-474 cells: the model estimated *k_p_* to be 3.3% lower, *k_d_* to be 2.7% higher, and *v* to be 0.9% lower when using the data from the first time point as the initial confluence, rather than the average of the first three time points. For MDA-MB-231 cells: the model estimated *k_p_* to be 21.3% higher, *k_d_* to be 1.5% higher, and *v* to be 5.3% higher when using the data from the first time point as the initial confluence, rather than the average of the first three time points.

*R2.10) Section 2.7: I understand the results but, as mentioned above, they suggest that the model needs revision. We are told you ‘found’ a relationship between kbys, the initial number of tumor cells and the initial glucose level – what other relationships did you try? Did you consider alternative, more physically-based models? You introduce 2 extra parameters (see equation (8)). How many extra parameters would be needed for a more physically-based model extension, where parameters do not depend on initial conditions? (eg introduce an additional variable for lactate levels, assume lactate production occurs at a rate proportional to glucose consumption, and lactate degradation at rate proportional to lactate levels, then cell death (your bystander term) is an increasing function of lactate levels – this extension would involve 2 additional parameters – did you try this?)*

Response:

We respectfully suggest it is both natural and practical to investigate the reliance of model parameters on initial conditions. If a relationship can be found between a given parameter and initial conditions, then that parameter can be assigned on an individual basis *via* an experimentally developed “look-up” table. Such an approach helps to “personalize” each prediction as these model parameters are determined by the initial condition of the experiment under investigation. Furthermore, the initial conditions are (by definition) frequently known (or, at least, bounded) at the beginning of the experiment so it provides a practical way to constrain model predictions. We systematically searched for relationships between the initial conditions (i.e., the initial confluence and initial glucose concentration) and all possible combinations of *k_p_*, *k_d_*, *v*, and *k_bys_* as global parameters or local parameters. We were able to find such a dependence on the initial conditions for *kbys* calibrated as a local parameter and with *k_p_*, *k_d_*, and *v* calibrated as global parameters. We have included a brief summary of these thoughts as additional motivation in Sections 2.5 and 2.7 of the revised manuscript.

As discussed in *R2.8*, we do not think revising the model to include the dependence of *k_bys_* on initial conditions is a better option because this relationship is developed based on fitting *kbys* as a local parameter. The effect of initial conditions on *k_bys_* is not known prior to model fitting and is most likely cell line specific (as suggested in the Discussion section). Additionally, as we don’t know which (or, if any) initial conditions would have an effect on *kbys* in a new cell line, a model that includes the dependence of *k_bys_* on initial conditions that we found in the BT-474 and MDA-MB-231 cell lines would be challenging to apply to other cell lines, thereby reducing the generalizability of the approach.

We absolutely agree there could be alternative, more physically-based models—in fact, there almost certainly are. However, such models will necessarily include additional parameters that cannot be calibrated given the data provided by the experiments described in this manuscript. For example, we did not assume that all cells use anaerobic glycolysis or that all cells use oxidative phosphorylation; rather, we include all possible states between complete oxidative phosphorylation and complete anaerobic glycolysis (please see response to *R1.3*). Therefore, if we introduce an additional variable to capture the temporal change in lactate concentration, we cannot simply assume lactate production occurs at a rate proportional to glucose consumption, because this implies a fixed fraction of the cells use anaerobic glycolysis. Assuming lactate degradation occurs at a rate proportional to lactate concentration implies that the fraction of cells use oxidative phosphorylation is proportional to the lactate concentration. Thus, the fraction of cells using oxidative phosphorylation would change with lactate concentration, which implies the fraction of cells using anaerobic glycolysis would also change and this contradicts the original assumption. For this modeling approach to work, we would need to introduce a varying fraction with two switching rates between anaerobic glycolysis and oxidative phosphorylation, at least one parameter for driving the switch depending on glucose and lactate concentrations, and a lactate consumption rate by oxidative phosphorylation. An extension like this would involve at least four additional parameters instead of two and would necessitate additional, time-resolved measurements. (Incidentally, a separate project in the lab is focused on developing optical methods to provide measurements of glucose and lactate concentration as a function of time; this is highly non-trivial.)

However, the reviewer’s point remains that other, more physically-based, modeling schema are definitely worth pursuing. Thus, in the revised Discussion section, we provide motivation for extending our model to explicitly incorporate additional (relevant) physical mechanisms and how they could be measured within an expanded experimental-computational framework.

*R2.11) Section 3.1: please consider presenting the results for the 2 cell types in a table – this*

*would be easier to digest than reading from the text.*

Response:

We appreciate the suggestion and have revised the manuscript accordingly as follows in Section 3.1.

Example time courses for the BT-474 cell line, with different initial confluences (i.e., seeding density) and four glucose concentrations are shown in Fig 2a-c. For wells with low initial confluence (Fig 2a), intermediate initial confluence (Fig 2b), and high initial confluence (Fig 2c), the percent change (mean ± 95% confidence interval) on the number of live cells from day 0 to day 4 with initial glucose concentrations of 0.2 mM, 0.5 mM, 2 mM, and 5 mM are shown in Table 2. The average number of live cells for each experiment at the end of day 4 was significantly different among the groups with different initial conditions (p < 1e-5).

Example time courses for the MDA-MB-231 cell line, with different initial confluences and four glucose concentrations, are shown in Fig 2d-f. For wells with low initial confluence (Fig 2d), intermediate initial confluence (Fig 2e), and high initial confluence (Fig 2f), the percent change on the number of live cells from day 0 to day 4 with initial glucose concentrations of 0.2 mM, 0.5 mM, 2 mM, and 5 mM are shown in Table 2. The average number of live cells for each experiment at the end of day 4 was significantly different among the groups with different initial conditions (p < 1e-5).)

**Table 2. Average percent change on the number of live cells from day 0 to day 4**

| Cell Line | Initial Confluence (%) | | Initial Glucose Concentration (mM) | | | |
| --- | --- | --- | --- | --- | --- | --- |
|  |  |  | 0.2 | 0.5 | 2 | 5 |
| BT-474 | Low | 23.8 ± 0.5 | -34.3 ± 12.3 | -6.5 ± 10.5 | +31.4 ± 8.4 | +35.7 ± 1.8 |
|  | Intermediate | 35.9 ± 1.8 | -63.7 ± 9.3 | -55.6 ± 3.1 | -10.4 ± 19.6 | +14.9 ± 8.3 |
|  | High | 51.7 ± 1.4 | -76.0 ± 1.0 | -76.3 ± 1.5 | -43.9 ± 12.4 | -17.6 ± 7.6 |
| MDA-MB-231 | Low | 36.9 ± 1.0 | -68.3 ± 7.9 | -47.7 ± 12.7 | +30.2 ± 13.5 | +32.9 ± 7.9 |
|  | Intermediate | 56.2 ± 1.4 | -63.7 ± 10.3 | -46.5 ± 10.6 | -1.8 ± 3.4 | +13.3 ± 2.1 |
|  | High | 71.9 ± 1.0 | -41.5 ± 11.6 | -33.6 ± 9.2 | -10.0 ± 3.2 | -1.6 ± 2.9 |

*R2.12) Section 3.2: Please can you clarify that no time course data on glucose levels was collected? It would be helpful to see what the model simulations predict for this hidden variable. It would be great, for example, to see how glucose levels are predicted to fall (and possibly how levels of waste products, e.g. lactate, increase) during the time course of the experiments.*

Response:

We apologize for the lack of clarity on this point. It is true that no data on glucose concentration as a function of time was collected. We have included, in the revised Supplemental Materials, predicted glucose levels over time for BT-474 cells (S6 Fig) with the same initial conditions as the examples presented in Fig 4, and for MDA-MB-231 cells (S7 Fig) with the same initial conditions as the examples presented in Fig 5. As mentioned in *R1.3*, our model includes all possible states between complete oxidative phosphorylation and complete anaerobic glycolysis. The lactate can be produced from pyruvate after glycolysis, but can also be converted to pyruvate and further utilized in oxidative phosphorylation. The lactate level may vary with the fraction of oxidative phosphorylation and anerobic glycolysis. Therefore, our current model is unable to predict the lactate levels under such assumptions. (We do note, though, that we are also working on a FRET reporter that would potentially allow us to measure glucose concentration over time; for a preliminary effort, see Yang *et al*. “*Towards Modeling of Tumor Cell Growth via FRET-guided Glucose Dynamics*.” 2019 Annual Meeting of the Biomedical Engineering Society; 1699; and Yeh *et al*. "*Deep learning enables rapid and robust analysis of fluorescence lifetime imaging in photon-starved conditions*." bioRxiv (2020)).

*R2.13) Section 3.2: are theta and Gmin global variables? How were they determined?*

Response:

Yes, both parameters are global variables and how they are determined are shown in Table 1.

*R2.14) Section 3.2 (line 390): do you have any idea why the error for dead cells is so large? (see earlier comments re model equations for possible explanations for this discrepancy)*

Response:

Please note that the large error is a percent error, the error relative to the number of dead cells. As shown in Table 3, the mean error, either across all timepoints or at the end of experiment, was < 2% for both live and dead cells in both cell lines. The percent error for dead cell is large simply because the number of dead cells is quite small.

*R2.15) Section 3.3: could you comment on the relative contributions that the different physical terms in the governing equations have on the growth dynamics (this is difficult to infer from the way that the parameter estimates are presented). For example, does cell proliferation dominate? How significant is the bystander effect? I appreciate that the importance of the different processes will change over time, but it would be great to have some sense of their relative contributions (perhaps at different time-periods – beginning, middle and end of experiments?).*

Response:

We very much appreciate this comment. The contributions from different physical terms in the governing equations depend on initial conditions and may change over time. In general, the contribution from logistic growth is dominant at the beginning and drops over time with the consumption of glucose. The contribution from death due to glucose depletion begins at time 0 and increases over time with the consumption of glucose. The contribution from death due to the bystander effect increases over time with the accumulation of dead cells. However, if there is sufficient glucose throughout the experiment, the contribution from logistic growth would remain dominant, with little contribution from death terms. We have included these comments in the revised Discussion section. Furthermore, we list several examples for the BT-474 cells (S3 Table) and MDA-MB-231 cells (S4 Table) in the revised Supplemental Materials. For each cell line, we list the relative contributions of each term at day 0, 2, and 4 for different initial confluences and three initial glucose concentrations (0, 1, and 10 mM) to show how the relative contributions vary over time with different initial conditions.

*R2.16) Section 4 (lines 593-609): these are good points, well made.*

Response:

We thank the reviewers for their complimentary remarks.

*R2.17) Section 5: what is the main conclusion/finding of the work? What new insight has been gained? Which model is most suitable? Why?*

Response:

We apologize for the lack of clarity on this point. The main finding of the work is the development of an experimental-mathematical approach capable of accurately predicting how glucose availability influences tumor cell proliferation. The model was also validated in two commonly studied breast cancer cell lines and can be applied to other tumor cell lines. The different relationships between *k_bys_* and the initial conditions found through model calibration suggested different mechanisms were involved in the bystander effect in these two breast cancer cell lines. The complete model characterized by Eqs. [1]-[5] featuring three global parameters (*k_p_*, *k_d_*, and *v*), and one local parameter dependent on initial conditions (*k_bys_*) was able to provide the best characterization of the data, as indicated by the lowest AICc value. We have revised the conclusion section of the manuscript to more clearly state these points.

*R2.18) General comment: the authors might consider using their model(s) to perform synthetic studies which could aid experimental design (see for example C Kreutz and J Timmer, FEBS Journal, 2009). Such studies might reveal the value of collecting glucose data at specific time points during the experiment, not necessarily at every time point.*

Response:

We appreciate this comment and have added a sentence and this reference on it in the Conclusion section as a possible application for future work.

1.	Kuhn M. Building Predictive Models in *R* Using the **caret** Package. J Stat Softw. 2008;28. doi:10.18637/jss.v028.i05

2.	Spitz MR, Hong WK, Amos CI, Wu X, Schabath MB, Dong Q, et al. A Risk Model for Prediction of Lung Cancer. JNCI J Natl Cancer Inst. 2007;99: 715–726. doi:10.1093/jnci/djk153

3.	Warburg O. On the Origin of Cancer Cells. Science. 1956;123: 309–314.

4.	Vander Heiden MG, Cantley LC, Thompson CB. Understanding the Warburg Effect: The Metabolic Requirements of Cell Proliferation. Science. 2009;324: 1029–1033. doi:10.1126/science.1160809

5.	Kenny PA, Lee GY, Myers CA, Neve RM, Semeiks JR, Spellman PT, et al. The morphologies of breast cancer cell lines in three-dimensional assays correlate with their profiles of gene expression. Mol Oncol. 2007;1: 84–96. doi:10.1016/j.molonc.2007.02.004

6.	Imamura Y, Mukohara T, Shimono Y, Funakoshi Y, Chayahara N, Toyoda M, et al. Comparison of 2D- and 3D-culture models as drug-testing platforms in breast cancer. Oncol Rep. 2015;33: 1837–1843. doi:10.3892/or.2015.3767

7.	Sullivan R, Graham CH. Hypoxia prevents etoposide-induced DNA damage in cancer cells through a mechanism involving hypoxia-inducible factor 1. Mol Cancer Ther. 2009;8: 1702–1713. doi:10.1158/1535-7163.MCT-08-1090

8.	Freeman SM, Abboud CN, Whartenby KA, Packman CH, Koeplin DS, Moolten FL, et al. The “Bystander Effect”: Tumor Regression When a Fraction of the Tumor Mass Is Genetically Modified. : 11.

9.	Li Bi W, Parysek LM, Warnick R, Stambrook PJ. In Vitro Evidence That Metabolic Cooperation Is Responsible for the Bystander Effect Observed with HSV tk Retroviral Gene Therapy. Hum Gene Ther. 1993;4: 725–731. doi:10.1089/hum.1993.4.6-725

10.	Asklund T, Appelskog IB, Ammerpohl O, Langmoen IA, Dilber MS, Aints A, et al. Gap junction-mediated bystander effect in primary cultures of human malignant gliomas with recombinant expression of the HSVtk gene. Exp Cell Res. 2003;284: 183–193. doi:10.1016/S0014-4827(02)00052-6

11.	Matono S, Tanaka T, Sueyoshi S, Yamana H, Fujita H, Shirouzu K. Bystander effect in suicide gene therapy is directly proportional to the degree of gap junctional intercellular communication in esophageal cancer. Int J Oncol. 2003;23: 1309–1315. doi:10.3892/ijo.23.5.1309

12.	Yang Y, Qin S-K, Wu Q, Wang Z-S, Zheng R-S, Tong X-H, et al. Connexin-dependent gap junction enhancement is involved in the synergistic effect of sorafenib and all-trans retinoic acid on HCC growth inhibition. Oncol Rep. 2014;31: 540–550. doi:10.3892/or.2013.2894

13.	Lyng FM, Seymour CB, Mothersill C. Production of a signal by irradiated cells which leads to a response in unirradiated cells characteristic of initiation of apoptosis. Br J Cancer. 2000;83: 1223–1230. doi:10.1054/bjoc.2000.1433

14.	Xu S, Wang J, Ding N, Hu W, Zhang X, Wang B, et al. Exosome-mediated microRNA transfer plays a role in radiation-induced bystander effect. RNA Biol. 2016 [cited 3 Apr 2020]. Available: https://www.tandfonline.com/doi/abs/10.1080/15476286.2015.1100795

15.	Azzam EI, Toledo SM de, Little JB. Oxidative metabolism, gap junctions and the ionizing radiation-induced bystander effect. Oncogene. 2003;22: 7050–7057. doi:10.1038/sj.onc.1206961
