# Supplementary material for: An experimental-mathematical approach to predict tumor cell growth as a function of glucose availability in breast cancer cell lines
Source: PLoS One. 2021 Jul 13;16(7):e0240765. doi: 10.1371/journal.pone.0240765 (PMC8277046; doi:10.1371/journal.pone.0240765)
Supplement: Attachment — Submitted filename: JY_Response to Reviewers.docx [file pone.0240765.s014.docx]

**Manuscript #PONE-D-20-30580R1**

We thank the reviewers for their careful consideration of our revised manuscript and for providing more constructive criticism. We have taken these comments seriously and have revised the manuscript accordingly. Detailed responses to each item are provided below. In our responses below, for clarity, we have referred to Review x, Comment y as ‘*Rx.y*’.

**Editor’s Comments:**

*Thank you for submitting your manuscript to PLOS ONE. After careful consideration, we feel that the reviewer comments were addressed and that the manuscript can be accepted pending the small suggested revisions below.*

Response:

We sincerely thank the Editor for their comments on the revision. As indicated below (and in the revised manuscript), we have worked hard to address each suggested revision.

**Specific review questions**

1. If the authors have adequately addressed your comments raised in a previous round of review and you feel that this manuscript is now acceptable for publication, you may indicate that here to bypass the “Comments to the Author” section, enter your conflict of interest statement in the “Confidential to Editor” section, and submit your "Accept" recommendation.

**Reviewer # 2:** All comments have been addressed.

Response:

We thank the reviewer for their support.

**Reviewer #3:** (No Response)

Response:

N/A

Reviewer 2 individual comments

*R2.0) The authors have done a good job of revising their manuscript in line with my comments and those of the other reviewer. As a result, I am happy to recommend it for publication.*

Response:

We sincerely thank the reviewer for their support.

*R2.1) I have only one minor request for further clarification: on page 15, the authors talk about 'reliance of the model parameters on the initial conditions' - what does this mean? that the model parameters are correlated with the initial conditions?*

Response:

We apologize for the lack of clarity on this point. Yes, we intended to investigate the correlation between the model parameters and the initial conditions. (The justification for model parameters depending on initial conditions can be found in response to *R2.8* in the initial ‘response to reviewers’ document and the revised manuscript.) We have rephrased the text on page 15 to provide more clarification on this point.

Reviewer 3 individual comments

*R3.0) The authors present a combined experimental and modeling approach to determine rates of proliferation, death, and glucose consumption for tumor cells, in varying glucose conditions. The paper achieves this goal, and the results are clearly presented. Though I did not review the first version, I have read the reviewers' comments and the authors' responses. I believe the authors have addressed the previous concerns.*

Response:

We sincerely thank the reviewer for their support.

*R3.1)* *The equations do not appear correctly, with operation signs (addition, multiplication) missing. I assume this will be corrected in the proofing stage.*

Response:

We apologize for this unfortunate situation and will work with the editor for correction in the proofing stage.

*R3.2)* *In the text, p values should be shown as numerical values (10^-4) rather than “1e-4”.*

Response:

We have changed the way we present the p values the revised manuscript to align with the reviewer’s suggestion throughout.

*R3.3) My primary major concern is in the presentation of the utility of the work. I would like to see some explanation about the impact and utility of the modeling work. In its current state, the paper does a great job in presenting the model and results. However, how others could take advantage of the work and use it to gain biological insight is missing. I feel this should be addressed in order to increase the impact of the work. How would the model do with a different cell line, say MCF7?*

Response:

We apologize for the lack of explanation for the impact and utility of the modeling work. The experimental-computational approach was developed and validated in two commonly studied breast cancer cell lines and is applicable to other cell lines. Given a new cell line, say MCF7, the same approach can be followed to estimate the proliferation rate, the death rate due to glucose depletion, and the consumption rate of glucose, as well as determining the correlation between the death rate due to the bystander effect and the initial conditions. Additionally, the framework allows for the prediction of the growth of a new tumor cell line given the initial conditions of glucose concentration and cell number. Thus, our experimental-mathematical approach allows for the systematic investigation of the response of different cell lines to glucose availability, thereby enabling the ability to quantitatively study the potential metabolism-related therapy. We have revised the Discussion section to address the impact and utility of the modeling work.

*R3.4) The abstract, intro, and methods emphasize the family of models and three approaches for calibration, but all of that (interesting stuff!) is relegated to a single table in the supplemental info (S1 Table). I would prefer to see a bit more description of those steps in the main text, as the work raises some interesting questions. For example, why do the model that neglects the bystander effect or the model that neglects death due to low glucose NOT fit the data well? These mechanistic questions are of interest, especially when considering the previous point - the utility of the work. If this is not the focus, but rather the goal is to show results from a single model, the intro and methods should be revised.*

Response:

We apologize for the lack of description of the other two members of the family of models and have added more details in the main text of the revised manuscript. The other two models did not perform as well as the model that included both death terms, as they yielded a significantly higher fitting error to the data. For model 1, the model that neglects the bystander effect, the death due to glucose depletion is not sufficient to capture all of the observed cell death, especially for cells seeded with high initial glucose and high initial confluence. For model 3, the model that neglects the death due to glucose depletion, the initiation of cell death cannot be explained when there are initially no dead cells and, therefore, perform worse and overestimate the death rate for cells with low glucose level and few dead cells at the beginning. We have added these comments to the revised Discussion section.
